# Short-Term Predictors for Weight Correction Success of the First Paediatric Weight Correction Programme in Children’s Clinical University Hospital in Riga

**DOI:** 10.3390/medicina55030075

**Published:** 2019-03-21

**Authors:** Jurgita Gailite, Dana Apela, Iveta Dzīvīte-Krišāne, Dace Gardovska

**Affiliations:** 1 Department of Pediatrics, Rīga Stradiņš University, Rīga LV-1007, Latvia; iveta.dzivite@bkus.lv (I.D.-K.); dace.gardovska@rsu.lv (D.G.); 2 Children’s Clinical University Hospital, Rīga LV-1004, Latvia; dana.apela@bkus.lv

**Keywords:** obesity, weight correction, overweight, children, multidisciplinary team

## Abstract

*Background and objectives:* The efficacy of a weight correction programme can be affected by such predictors as the number of contact hours, gender, age, baseline weight, parental weight status, psycho-emotional status, insulin resistance, and socioeconomic status. The aim of this current study was to evaluate the overall efficacy of the Weight Correction Programme at Children’s Clinical University Hospital, and explore the impact of the probable predictors. We evaluated the efficacy depending on gender, age, parental weight status, signs of depression, baseline body mass index z-score (z-BMI), and baseline waist circumference. *Materials and Methods:* The data were gathered from medical records. The inclusion criteria were as follows: Entered the programme by 13 June 2017, at least five years old, follow-up data available. All the respondents were divided into two age groups: <10 years old and ≥10 years old. *Results:* The study included 181 respondents. They were 5 to 17 years old on the first day of participation in the Weight Correction Programme. Results indicated that 117 (65%) patients managed to reduce z-BMI and 69 (38%) patients achieved clinically significant reduction of z-BMI. Boys had four times higher odds (odds ratio (OR) = 4,22; CI 1.37–13.05; *p* = 0.012) to reduce their z-BMI by at least 0.20 units than girls. The respondents of the older age group (≥10 years) had a better chance to reduce z-BMI than the younger ones (OR = 11,51; CI 2.04–64.83; *p* = 0.006). The odds to reduce z-BMI were lower by 7% for every extra cm of waist circumference (OR = 0.93; CI 0.88–0.99; *p* = 0.014) for reducing z-BMI. The follow-up time was also a positive predictor, and with every month the odds for clinically significant z-BMI reduction increased by 7% (OR = 1.07; CI 1.00–1.15; *p* = 0.047). The parental weight status, signs of depression, and baseline z-BMI were not significant predictors. *Conclusions:* More than half of the patients of the respondents managed to reduce their z-BMI. Female gender, younger age, and larger waist circumference were negative predictors.

## 1. Introduction

The prevalence of overweight children varies a lot across European countries, from 14.8% in Tajikistan to 37.3% in Greece. According to the World Health Organisation (WHO), 21.5% of children at least five years old were overweight in Latvia in 2016 [1]. Moreover, in Latvia, the prevalence of excess weight is quite stable for children at age seven [2], but tends to increase among adolescents [3]. The WHO defines overweight as a standardized body mass index z-score (z-BMI) above +1 SD for children at least five years old [4].

Most children retain their excess weight into adulthood if no intervention is done [4]. The main, and often only, available kind of intervention is a nonsurgical, nonpharmacological therapy in a multidisciplinary weight correction programme [5]. The treatment should target the patient’s diet, physical activity, and psychological problems, as well as parenting issues [6]. Thus, multidisciplinary programmes provide family-based behavioural treatment. The obvious aim of weight correction programmes is reducing relative weight (body mass index (BMI), z-BMI, % above 95th percentile), improving lipid and insulin levels, and delaying complications. Nevertheless, participating in such a programme improves psychosocial functioning as well [6]. Pharmacological treatment is considered to be an intermediate step between a multidisciplinary approach and surgery. There is only one drug—metformin—available for adolescents in Europe. Nevertheless, its effects are well studied for diabetes treatment, but they are not so well understood and predictable for overweight adolescents without diabetes [7]. Bariatric surgery is only acceptable in case of morbid obesity after puberty and if the multidisciplinary approach has already been tried and is going to be continued after surgery [8].

When Grossman et al. analysed those weight correction programmes that showed statistically significantly better results than the control group with no intervention, it turned out that the mean reduction of z-BMI after a year of treatment was at least 0.11 units [9,10]. Nevertheless, Muhlig et al. observed a wide range of z-BMI mean reductions across multidisciplinary programmes—from 0.05 to 0.42 units. The follow-up visits close to the one-year period were taken into account [11]. Another way to evaluate the efficacy of a weight correction programme is to look at the percentage of patients that have managed to reduce their z-BMI. Dalla Valle et al. observed a z-BMI reduction in 60% of all respondents after 12 months of treatment [12]. A better way to look at the results is to assess the percentage of patients with clinically significant z-BMI reduction (e.g., by at least 0.20 units) [9,10]. Shalitin et al. observed clinically significant results in 38.5% of respondents after a two-year long treatment [13]. Wiegand et al. reported a z-BMI reduction by at least 0.20 units in 41% of respondents after a year of treatment [14].

There are lots of different possible predictors studied around the world that may affect the success of paediatric weight correction programmes. The USA meta-analysis reveals the number of contact hours being the main predictor—usually at least 26 contact hours per year are needed for a programme to be effective [10]. Insulin resistance and factors associated with it, such as acanthosis nigricans and central adiposity (great waist circumference), are known to be negative predictors [12,15]. Positive predictors for a child’s weight correction are concurrent parental weight loss [16], better motivation [12], and better socioeconomic status [15]. The evidence about other predictors is not so consonant. Age [12,17], gender [14,18], and baseline relative weight [15,16] are described as positive and negative predictors.

The first paediatric multidisciplinary weight correction programme in Latvia has been working since 26 August 2014. It takes place at Children’s Clinical University Hospital (CCUH) in Riga.

This study is the first to assess the efficacy of the CCUH Weight Correction Programme. Our aim was to evaluate the possible positive and negative predictors for weight correction success in overweight children.

## 2. Materials and Methods

### 2.1. The Weight Correction Programme

The main inclusion criterion of the multidisciplinary weight correction programme at CCUH was alimentary (exogenous) overweight/obesity, but motivation was considered as well. Overweight patients, aged 5–17 years old, were included in the weight correction programme if their parents were ready to attend the programme together with their child. The exclusion criteria were: Genetic syndromes, endocrine pathologies, therapy with biologic medicaments for rheumatologic diseases, mental retardation, movement restrictions, seizures. At first the patient stayed in day stationary for two days. The endocrinologist, rehabilitologist, nutritionist, physiotherapist, and psychologist examined and evaluated the child during the first day. An individual management plan was made, and specific targets were set for each patient during the second day. Further on, the programme was carried out in an outpatient setting. According to an individual plan each patient visited each of the specialists at least three times during the following year. This meant at least 20 contact hours over a one-year period. The last visit of this period was the moment when the achieved results were assessed, and a further plan of observation and treatment was formulated.

### 2.2. Study Population

The data were gathered from Latvian CCUH electronic databases, Andromeda and Saule, as well as from ambulatory cards from 1 December 2017 to 31 March 2018. There were 303 patients that entered the Weight Correction Programme at CCUH from 26 August 2014 to 13 June 2017. During the data gathering period, 181 patients already had anthropometric data (weight and height) available from at least one follow-up visit. Thus, there were 181 patients included in this cross-sectional study. Whenever there were data available from more than one follow-up visit, the latest anthropometric measurements from the last visit were considered to access the success.

### 2.3. Measures

Such anthropometric parameters as height, weight, and waist circumference were measured on the first day of the programme and during the follow-up visit with the endocrinologist. The weight and height data were transformed into body mass index z-score (z-BMI) using the WHO Anthro Plus programme.

The respondents were divided into two groups according to their age on the first day of the programme: <10 years old and ≥10 years old.

The information about parental weight status (is or is not overweight) had been documented on the first day of the programme. Thus, all respondents were divided into four groups: Both parents with no excess weight, only mother overweight, only father overweight, both parents overweight.

The Children Depression Inventory (CDI) had been used on the first day of the programme to assess for signs of depression [19]. The cut-off point of 13 points was used for CDI results when dividing the respondents into two groups.

The information about respondents’ birth weight was also available. Respondents were divided into two groups according to birth weight: <4 kg and ≥4 kg.

### 2.4. Statistical Analysis

The IBM SPSS Statistics v.25.0 (IBM, Armonk, NY, USA) was used for data analysis. The normality of data distribution was evaluated by the Shapiro–Wilk test. The Wilcoxon signed-rank test was used to compare the baseline and follow-up median z-BMI. The Mann–Whitney test was used for comparison of two independent samples. The effect size was calculated as a ratio of Wilcoxon’s Z value by the square root of number of observations. The main outcome measures were the number of patients that had reduced their z-BMI and the number of patients with clinically significant z-BMI reduction (by at least 0.20 units). The logistic regression was used for evaluating the significance of each of the available possible predictors: Parental weight status, gender, birth weight, age, baseline z-BMI, follow-up time, waist circumference, signs of depression. Even those predictors that were not significant in unifactorial logistic regression analysis were included in the multifactorial logistic regression. The results with *p* < 0.05 were considered significant.

### 2.5. Ethical Approval

The study was allowed by the CCUH, as well as by the Ethics Committee of Riga Stradins University (Ethical code Nr. 42, date of approval 30.11.2017.)

## 3. Results

We included 181 respondents (47% boys) with a median age of 12 (Interquartile range (IQR) 10;14) years. Most of the respondents (82%) were at least ten years old on the first day of the programme. The median z-BMI on the first day was 2.79 (IQR 2.33;3.14). The median waist circumference on the first day was 98 (IQR 90;107) cm. The anthropometric data according to gender and age groups from the first day of participation and the last follow-up visit are described in the Table 1. The median follow-up time for the whole sample was 8 (IQR 4;15) months, moreover, 75 (41%) respondents’ follow-up time was less than 6 months. There was a significant decrease of median z-BMI to 2.65 (IQR 2.18;3.07) units, *p* < 0.001. The effect size was 0.26. The median change of z-BMI was −0.09 (IQR −0.31;+0.07) units. Moreover, 117 (65%) patients managed to reduce their z-BMI, and 69 (38%) patients achieved clinically significant reduction of z-BMI (by at least 0.20 units).

When the reduction of z-BMI was chosen as the main outcome in the logistic regression, none of the possible predictors was statistically significant in the unifactorial analysis. Nevertheless, the age and waist circumference became significant in the multifactorial logistic regression. The results are shown in Table 2.

If the clinically significant reduction of z-BMI (by at least 0.20 units) was chosen as a main outcome, the gender was a significant predictor in the unifactorial regression, as well as in the multifactorial regression. Baseline z-BMI was significant in the unifactorial regression but became insignificant in a multifactorial regression. On the contrary, the follow-up time became significant in the multifactorial regression. The results are shown in Table 3.

## 4. Discussion

Though the median z-BMI reduction does not reach the 0.11 unit level [9,10], the statistically significant reduction of the median z-BMI, as well as the negative median z-BMI change, shows that the Weight Correction Programme at Latvian Children’s Clinical University Hospital s beneficial. The minimum of provided contact hours over a year in this programme was 20 h, which is less than the recommended 26 h by the US Preventive Services Task Force [10]. Moreover, about 40% of patients had participated less than half a year by the follow-up visit included in the data analysis. Therefore, this study assessed the short-term results with no information about such long-term z-BMI patterns as rapid success/maintenance, delayed success/maintenance, cycling, and initial success/rebound [14,20].

More than half of the patients had already managed to reduce their z-BMI. This result was quite similar to a study by Dalla Valle et al. [12]. Furthermore, the percentage of patients with a clinically significant reduction of their z-BMI in our study was the same as reported by Shalitin et al. [13].

The median baseline z-BMI of our sample was higher than mentioned in many other publications [13,15,16,17,21]. There was even a lower mean baseline z-BMI value than in our sample described in The Evidence Report and Systematic Review for the US Preventive Services Task Force [10]. A child’s high z-BMI was a positive predictor for parents to recognize their child was overweight [2,22,23].

The male gender was a positive prognostic factor for achieving clinically significant z-BMI reduction in the Weight Correction Programme at CCUH. This was consistent with the results of Wiegand et al. [14], but inconsistent with Walker et al. [24]. In their report, Wiegand et al., used z-BMI as we do, but Walker et al. used BMI. Thus, it is possible that Walker et al. may have underestimated the results of boys as their BMI physiologically grows more than female BMI. Using z-BMI is a better way of evaluating the results of a paediatric weight correction programme than using simple BMI [25].

The best way for stratifying the respondents would be by Tanner’s stage [26]. Unfortunately, we had no data available about the pubertal status of each respondent at the time of each visit. Therefore, we stratified the sample into two groups with 10-years-old being the border. We presumed those younger than 10 years as prepubertal children, but the older ones as pubertal or postpubertal adolescents.

The multifactorial regression analysis showed age as a significant predictor for reduction of z-BMI. At least part of this impact can be explained by a different approach to younger patients and less intense management. It is recommended to target an adult as the agent of change for weight correction in children 6–11 years old. On the contrary, the adolescents themselves are the implementers of weight correction recommendations [27]. Even if there was no intensive weight correction result observed for the younger ones, their participation in such a programme helped improved children’s health, self-esteem, and future prognosis for weight correction and avoiding cardiometabolic health issues [6,27].

The Weight Correction Programme at CCUH did not include children with insulin resistance. The only marker associated with insulin resistance that we used as a possible predictor was the waist circumference. As in other publications [15], higher waist circumference turned out to be a negative predictor for reducing z-BMI in our study.

As in the study by Shalitin et al. [13] we did not manage to prove the impact of parental weight status upon the odds to reduce a child’s z-BMI. The explanation could be the fact that we had access only to the first day data about the parental weight status. Evidence shows that the concurrent change of parental weight status is a significant predictor for a child’s weight correction success [16], as well as the change of a child’s weight outside the weight correction programme [28]. Thus, tracking the contemporary change of parental weight would be important for evaluating the real impact of parental weight.

The restrictions of this study were connected to the design of the study, as well as the complexity of the subject. The Weight Correction Programme at CCUH provides an individually adjusted management plan. Thus, the intensity of therapy varies among the patients, though it is declared that everyone gets at least 20 contact hours during a year. The specific inclusion criteria for the Weight Correction Programme at CCUH states that only motivated overweight and obese children and adolescents can participate. The motivation, itself, is a predictor of weight correction status [17]. Thus, it is hard to attribute the results of this study to the whole population of children with excess weight in Latvia. The data gathering from medical records lets us use only the data documented in ambulatory and stationery cards. That is why there was no information available about such possible predictors as: The number of visits with all of the specialists [29], parental contemporary weight change [28], simultaneous changes in depression signs, parental education [30], the number and gender of siblings and the birth order [31], socioeconomic status [15], parental marital status [32], Tanner’s stage [27]. Not all patients had documented information about birth weight, waist circumference, parental weight status, and CDI results. At the time of data gathering, only 55 (30%) children had anthropometric data available after at least one-year follow-up time. We analysed only the baseline and last visit anthropometric data—no information about the z-BMI fluctuations during the participation in the weight correction programme was included. Nevertheless, the follow-up time of our respondents was quite short. Thus, most likely there would be no z-BMI fluctuation patterns described by Wiegand et al. [14].

## 5. Conclusions

More than half of patients of the Weight Correction Programme at Latvian Children’s Clinical University Hospital have managed to reduce their z-BMI. More than one third of patients have achieved clinically significant improvement.

The participants of CCUH Weight Correction Programme have a higher median baseline z-BMI than described in literature.

Male gender, older age, and lower waist circumference are positive predictors for weight correction success. A more intense approach is needed for female patients as well as for children under the age of 10 years, and for those with central adiposity.

According to the literature, this weight correction programme could benefit from increasing the number of contact hours per year.

## Figures and Tables

**Table 1:** The anthropometric data for each gender and age group.

	Gender		*p* Value	Age Group		*p* Value
	Boys	Girls		<10 Years	≥10 Years	
*n* (%)	85 (47)	96 (53)		33 (18)	148 (82)	
z-BMI_1_, median (IQR), SD	2.96	2.63	<0.001	2.96	2.77	0.095
	(2.62;3.28)	(2.20;2.95)		(2.41;3.74)	(2.30;3.10)	
WC_1_, median (IQR), cm	99	96	0.014	87	99	<0.001
	(92;108)	(88;103)		(82;94)	(92;108)	
z-BMI_2_, median (IQR), SD	2.82	2.52	0.005	2.91	2.60	0.021
	(2.28;3.21)	(2.09;2.91)		(2.30;3.37)	(2.15;3.00)	
Change of z-BM I *, median (IQR), SD	−0.15	−0.06	0.028	−0.13	−0.09	0.810
	(−0.41;0.04)	(−0.25;0.10)		(−0.36;0.15)	(−0.27;0.06)	
Relative change of z-BMI **, median (IQR), SD	−4.4	−2.4	0.039	−3.3	−3.5	0.446
	(−15.3;1.4)	(−8.1;4.4)		(−11.9;5.4)	(−11.1;1.9)	
Patients with reduction of z-BMI, n (%)	61 (72)	56 (58)	0.059	18 (55)	99 (67)	0.180
Patients with reduction of z-BMI by at least 0.20 units, n (%)	39 (46)	29 (30)	0.030	13 (39)	55 (37)	0.811

z-BMI_1_—z-scores (standard deviation scores) of body mass index on the first day of participation; IQR—interquartile range; SD—standard deviations; WC_1_—waist circumference on the first day of participation; z-BMI_2_— z-scores (standard deviation scores) of body mass index at the follow-up visit; *—estimated using the following equation: z-BMI_2_—z-BMI_1_; **—estimated using the following equation: (z-BMI_2_—z-BMI_1_)/z-BMI1.

**Table 2:** The predictors of reducing z-BMI.

Factors	OR	95% CI	*p*	Adjusted OR	95% CI	*p*
Parental excess weight						
Both parents	1.51	0.66–3.49	0.331	2.14	0.53–8.74	0.287
Only father	1.16	0.46–2.98	0.751	0.88	0.22–3.54	0.861
Only mother	0.50	0.22–1.17	0.109	0.74	0.22–2.44	0.619
(Reference: none overweight)						
Gender: boys	1.74	0.93–3.25	0.082	1.74	0.62–4.88	0.296
(Reference: girls)						
Birth weight ≥4 kg	0.93	0.45–1.92	0.835	0.72	0.21–2.47	0.606
(Reference: <4 kg)						
Age: ≥10 years	1.68	0.78–3.62	0.183	**11.51**	**2.04–64.83**	**0.006**
(Reference: <10 years)						
z-BMI_1_	1.45	0.95–2.19	0.085	2.15	0.81–5.68	0.123
(step: 1 unit)						
Follow-up time	0.99	0.95–1.04	0.792	1.02	0.96–1.09	0.472
(step: 1 month)						
Waist circumference	1.00	0.98–1.03	0.713	**0.93**	**0.88–0.99**	**0.014**
(step: 1 cm)						
Signs of depression (Reference: no signs of depression)	1.53	0.67–3.51	0.314	2.17	0.72–6.67	0.168

OR—odds ratio; CI—confidence interval; z-BMI_1_ —z-scores (standard deviation scores) of body mass index on the first day of participation.

**Table 3:** The predictors of reducing z-BMI by at least 0.20 units.

Factors	OR	95% CI	*p*	Adjusted OR	95% CI	*p*
Parental excess weight						
Both parents	1.75	0.80–3.83	0.159	3.068	0.77–12.21	0.112
Only father	1.13	0.45–2.83	0.789	0.412	0.08–2.25	0.306
Only mother	0.69	0.28–1.70	0.422	0.865	0.23–3.30	0.832
(Reference: none overweight)						
Gender: boys	**1.96**	**1.07–3.60**	**0.031**	**4.22**	**1.37–13.05**	**0.012**
(Reference: girls)						
Birth weight ≥4 kg	0.55	0.25–1.19	0.129	0.46	1.84–0.12	0.274
(Reference: <4 kg)						
Age: ≥10 years	0.95	0.85–1.07	0.411	5.16	0.77–34.56	0.091
(Reference: <10 years)						
z-BMI_1_	**1.83**	**1.21–2.76**	**0.004**	2.33	0.75–7.20	0.143
(step: 1 unit)						
Follow-up time	1.02	0.98–1.07	0.336	**1.07**	**1.00–1.15**	**0.047**
(step: 1 month)						
Waist circumference	1.00	0.98–1.03	0.727	0.94	0.87–1.01	0.088
(step: 1 cm)						
Signs of depression (Reference: no signs of depression)	1.53	0.67–3.51	0.314	1.15	0.38–3.49	0.799

OR—odds ratio; CI—confidence interval; z-BMI_1_—z-scores (standard deviation scores) of body mass index on the first day of participation.
